# Quantification of Cell-Free DNA in Red Blood Cell Units in Different Whole Blood Processing Methods

**DOI:** 10.1155/2016/9316385

**Published:** 2016-09-27

**Authors:** Andrew W. Shih, Vinai C. Bhagirath, Nancy M. Heddle, Jason P. Acker, Yang Liu, John W. Eikelboom, Patricia C. Liaw

**Affiliations:** ^1^Department of Pathology and Molecular Medicine, McMaster University, Hamilton, ON, Canada; ^2^McMaster Centre for Transfusion Research, McMaster University, Hamilton, ON, Canada; ^3^Department of Medicine, McMaster University, Hamilton, ON, Canada; ^4^Thrombosis and Atherosclerosis Research Institute, McMaster University, Hamilton, ON, Canada; ^5^Centre for Innovation, Canadian Blood Services, Hamilton, ON, Canada; ^6^Department of Laboratory Medicine and Pathology, University of Alberta, Edmonton, AB, Canada; ^7^Centre for Innovation, Canadian Blood Services, Edmonton, AB, Canada

## Abstract

*Background*. Whole blood donations in Canada are processed by either the red cell filtration (RCF) or whole blood filtration (WBF) methods, where leukoreduction is potentially delayed in WBF. Fresh WBF red blood cells (RBCs) have been associated with increased in-hospital mortality after transfusion. Cell-free DNA (cfDNA) is released by neutrophils prior to leukoreduction, degraded during RBC storage, and is associated with adverse patient outcomes. We explored cfDNA levels in RBCs prepared by RCF and WBF and different storage durations.* Methods*. Equal numbers of fresh (stored ≤14 days) and older RBCs were sampled. cfDNA was quantified by spectrophotometry and PicoGreen. Separate regression models determined the association with processing method and storage duration and their interaction on cfDNA.* Results*. cfDNA in 120 RBC units (73 RCF, 47 WBF) were measured. Using PicoGreen, WBF units overall had higher cfDNA than RCF units (*p* = 0.0010); fresh WBF units had higher cfDNA than fresh RCF units (*p* = 0.0093). Using spectrophotometry, fresh RBC units overall had higher cfDNA than older units (*p* = 0.0031); fresh WBF RBCs had higher cfDNA than older RCF RBCs (*p* = 0.024).* Conclusion*. Higher cfDNA in fresh WBF was observed compared to older RCF blood. Further study is required for association with patient outcomes.

## 1. Introduction

Transfusion of red blood cells (RBCs) is one of the most widely used therapies in clinical medicine. In Canada, approximately 1.5 million transfusions were given each year from 2006 to 2012, with the majority being red blood cells (RBC). Emerging data suggests that there is variability in RBC product quality depending on the method used to process the whole blood donations and the duration of RBC storage [1]. Since 2008, Canadian Blood Services (CBS) produce RBCs using two different methods in an approximately 1 : 1 ratio, the red cell filtration (RCF) (also called buffy coat) method and the whole blood filtration (WBF) method [2]. In the RCF method, whole blood is held at room temperature for a maximum 20 hours before separation into platelets, plasma, and RBCs and red cell leukoreduction occurs at room temperature. In the WBF method, the whole blood is cooled within 8 hours of collection to 4°C and then leukoreduced in the cold and processed into plasma and RBCs any time within 72 hours of the collection.

Differences in processing method may have a negative impact on patient outcomes [1, 3]. A retrospective review of over 23,000 patients receiving approximately 92,000 RBC transfusions over a six-year period in three tertiary care centers demonstrated higher in-hospital mortality in patients who received WBF products with a shorter storage duration (less than 8 days) compared to patients who received RCF products with a longer storage duration [4]. One difference between the two methods of whole blood processing is the timing and temperature of leukoreduction. In blood products that have not undergone leukoreduction, there are significant levels of cfDNA and associated histones that increase with time [5]. Both are released in the form of neutrophil extracellular traps (NETs) by neutrophils in the presence of microbial or inflammatory stimuli [6]. cfDNA activates coagulation via the contact pathway [7]. Interactions with platelets and neutrophils can result in microvascular thrombosis, leading to tissue hypoxia and endothelial damage. Histones activate platelets [8], induce neutrophil accumulation in organs [9], and cause endothelial cell toxicity [10]. cfDNA can also be released from mitochondria, in which case it is not associated with histones. Mitochondrial DNA (mtDNA) has similar procoagulant and platelet-stimulating potential as nuclear cfDNA but also has distinct proinflammatory properties [11, 12]. In animal models, reducing NETs with a DNA-digesting enzyme or inhibiting NETs with anti-histone antibodies results in improved survival in an animal model of transfusion-associated lung injury [13], and neutralizing histones with antibodies can rescue mice from lethal sepsis [14]. In humans, circulating cfDNA levels have been associated with deep vein thrombosis, increased risk of mortality in septic patients [15], increased severity in trauma patients, and thrombosis in cancer patients [16]. Thus, the cfDNA released from white blood cells is potentially harmful.

We hypothesized that the delay in leukoreduction in blood processed by the WBF method may lead to higher amounts of cfDNA released from leukocytes, thereby potentially explaining the observed association with increased mortality when the WBF product is transfused. We also hypothesized that DNases in blood may contribute to degradation of DNA over time and thus fresh blood will have higher amounts of cfDNA compared to older blood. To test these hypotheses, we measured cfDNA in RBC products and correlated these levels with the method of whole blood processing and duration of storage.

## 2. Materials and Methods

### 2.1. Sample Collection

Approximately 5 mL was sampled from packed RBC units in the Transfusion Medicine laboratory at the McMaster site of Hamilton Health Sciences using a sterile docking device. Samples were consecutively collected to meet a 1 : 1 ratio of fresh blood (defined as having a storage time of 14 days or less) or older (storage time greater than 14 days) blood. We utilized a cutoff of 14 days or less for fresh blood as it was the most common definition utilized for fresh blood [17]. The samples were immediately spun at 1700 g for 10 minutes, and the supernatant was aliquoted and frozen at −80°C. DNA from 200 *μ*L of thawed supernatant was extracted into 200 *μ*L of AE buffer (elution buffer) using the DNeasy Blood and Tissue Kit (Qiagen, Hilden, Germany) as per the manufacturer's directions.

Anonymized data including product number and storage duration were recorded from the unit at the time of sampling. The unique product numbers were documented and sent to Canadian Blood Services who provided the method of processing for each unit. The protocol was approved by the Hamilton Integrated Research Ethics Board and the Research Ethics Board at Canadian Blood Services.

### 2.2. Measurement of Cell-Free DNA Concentration

DNA concentration was determined by spectrophotometry, with concentration of DNA measured with UV absorbance at 260 nm on an Eppendorf Biophotometer Plus (Eppendorf, Hamburg, Germany). The PicoGreen assay (Life Technologies, Carlsbad, CA) was performed as per the manufacturer's directions, where a smaller volume of 100 *μ*L per sample was used and read in 96-well opaque black plates.

### 2.3. Statistical Analysis

Statistical analysis was performed using computer software (SAS Version 9.3, Cary, North Carolina). Descriptive analyses of continuous variables were reported as mean and standard deviations. General linear regression models were conducted to determine the association between age of blood and whole blood processing method independently with cfDNA concentration by spectrophotometry or PicoGreen. Age of blood was analyzed both as a dichotomous variable (with fresh blood denoted as being stored for 14 days or less and older blood denoted as being stored for 15 days or more) and a continuous variable. The interacting effect between age of blood as a dichotomous variable and processing method on cfDNA measurements was also assessed in a separate regression model with an interaction term. Bonferroni adjustment was used for multiple comparisons and results were considered significant at *p* values of less than 0.025. As a secondary analysis, we also assessed if longer duration before leukoreduction predicted higher levels of cfDNA.

### 2.4. Sample Size Calculation

In another study analyzing cfDNA levels in stored blood [5], healthy control donors had a mean plasma cfDNA level of approximately 50 ng/mL. The mean level of cfDNA found in nonleukoreduced RBC units stored for 42 days was approximately 100 ng/mL (SD 30 ng/mL), an increase of 100%. We hypothesized that cfDNA will increase by approximately 50% in WBF units compared with RCF units. Calculating sample size using a two-sided test, an alpha of 0.05, and a desired power of 0.80, the sample size for each group was determined to be 48 samples. The method by which a unit of blood is processed is not known when the RBC arrives at the hospital; however, the ratio of RCF and WBF units produced by Canadian Blood Services is approximately 1 : 1; hence, we estimated that sampling 120 RBC units would provide a 95% probability of having at least 48 samples by each production method.

## 3. Results

120 units were sampled in total, with 60 being fresh units (≤14 days of storage duration) and 60 being older units. Of the 60 fresh units, 48 (80%) had a storage duration of less than 8 days. After the method of processing was provided by Canadian Blood Services, it was determined that 73 units were made with the RCF method and 47 were made by the WBF method.

### 3.1. WBF Processed RBC Units Had Higher cfDNA Compared to RCF Units Processed Units by PicoGreen

To test our hypothesis that WBF processed RBC units have higher amounts of cfDNA, we compared cfDNA concentrations in WBF and RCF RBC units. cfDNA was significantly higher in WBF RBCs compared to RCF units when quantified by PicoGreen (1.08 ± 0.90 ng/mL versus 0.50 ± 0.77 ng/mL, *p* = 0.0010) (Table 1). When the interaction between storage duration and processing method was considered for cross-comparisons, fresh WBF RBCs had significantly higher levels of cfDNA than fresh RCF RBCs as measured by PicoGreen (1.16 ± 1.14 ng/mL versus 0.37 ± 0.77 ng/mL, *p* = 0.0093) (Table 2). No significant difference was seen between cfDNA in WBF and RCF RBCs when measured by spectrophotometry (*p* = 0.088), although the absolute values were concordant with the findings by PicoGreen. Similar results were obtained when age of blood was analyzed as a continuous variable. In this analysis, there was a trend towards increased cfDNA measured by spectrophotometry in WBF compared to RCF RBCs overall (*p* = 0.063).

### 3.2. Fresh RBC Units Had Higher cfDNA Compared to Older RBC Units by Spectrophotometry

Fresh RBCs were found overall to have a significantly higher concentration of cfDNA compared to older RBCs using spectrophotometry (3.77 ± 1.66 *μ*g/mL versus 3.02 ± 1.44 *μ*g/mL, *p* = 0.0031) (Table 3). This association strengthened when age of blood was analyzed as a continuous variable (*p* = 0.00066). When the interaction between storage duration and processing method was considered, fresh WBF RBCs had significantly higher cfDNA compared to older RCF RBCs (*p* = 0.024) (Table 2). No significant difference overall was seen when comparing cfDNA quantified by PicoGreen in fresh compared to older units (*p* = 0.33), even when age of blood was analysed as a continuous variable (*p* = 0.39).

We examined whether longer time to leukoreduction predicted higher levels of cfDNA but did not demonstrate an association, possibly because of relatively small numbers of products for which data was available (*n* = 32).

## 4. Discussion

Differences in RBC production method could affect clinical outcomes, with retrospective data suggesting that fresh blood produced by the WBF method could be associated with an increased risk of in-hospital mortality [18]. Retrospective studies of this nature could always be susceptible to confounding; however, if this association is true, biological mechanisms that could explain this finding need to be explored. In this study, we investigated the possibility that cfDNA levels could vary in different RBC products and possibly be an explanation for the clinical observations that have been observed. We found that RBC products produced by the WBF method have higher levels of cfDNA than products produced by the RCF method. We also found that fresh RBC products have higher levels of cfDNA than older products. Hence, the results of this study are consistent with the hypothesis that cfDNA in transfused RBC products could have an impact on patient outcomes.

The results of our study are consistent with other data showing that the in vitro quality of RBCs varies by method of processing and storage duration. RBCs processed by WBF have qualitative differences from RBCs processed by RCF such as higher residual plasma [19], smaller red cell microvesicles [20], higher amounts of hemolysis at expiry, lower ATP levels [21], and higher MCV at expiry [1]. A previous study demonstrated higher mtDNA with RBCs processed by WBF, where our testing for cfDNA encompasses mtDNA as well as nuclear cfDNA [21]. mtDNA may potentially have immunomodulatory properties [11], where nuclear cfDNA may be more procoagulant [7]. The clinical significance of these differences is unclear.

There are several limitations to our study. While we demonstrated the correlation between WBF processed blood with a shorter duration of storage and increased cfDNA compared to RCF blood with a longer duration of storage, we cannot conclusively state that methods of blood preparation and storage duration are causative for our findings. How NETosis is affected by specific processing variables such as temperature, centrifugation force, extraction method, filter design, blood bags used, storage solution, and anticoagulant was not studied in our exploratory analysis. Other variables such as donor characteristics were also not assessed and could also be playing a role in patient outcomes [22]. However, consecutive sampling of units avoids selection bias and our sample size was appropriately conservative to account for potential variation amongst units. We also could not prove that cfDNA is linked to the pathobiology of fresh WBF units causing harm as we did not prospectively follow patients transfused with these units. A prospective study to link clinical outcomes would require a much larger sample size to demonstrate a conclusive effect.

A second issue is the difficulty in measurement of cfDNA. We found poor correlation between cfDNA levels measured by the two methods. This could be due to differences in sensitivities and specificities of each assay, where PicoGreen is specific for double-stranded DNA. The significance of single-stranded DNA and double-stranded DNA is unknown. Both assays are potentially affected by protein contamination, most notably with the spectrophotometry method. We also observed a high degree of variance with cfDNA measurements, where donor factors, specific parameters within whole blood processing, or poor precision in current methods of cfDNA measurement could be potential contributors. However, the higher levels of cfDNA in WBF and in fresher products by both methods, accounting for multiple tests of significance, suggest robustness of this finding. We did not perform testing for histones or nucleosomes. Our results do not differentiate between nuclear and mitochondrial DNA.

Our study has several strengths. We sampled a relatively large number of units in relation to our calculated sample size and explored the effect of duration of storage of blood as well as the method of whole blood processing. Concordance of findings in the quantification of cfDNA increases the robustness of our conclusion of increased cfDNA in fresh compared to older blood and in WBF compared to RCF blood. Our finding that older products had less cfDNA is consistent with results from recent randomized trials comparing fresh to standard issue (older) blood, as these studies have not shown that fresh blood is superior [23–25], with some trials suggesting a trend towards harm with fresher blood [18, 24, 26].

A prospective study with patients transfused blood produced via different methods of whole blood processing could link adverse patient outcomes to sampled transfused blood products and recipients. This would allow for testing of cfDNA levels as well as other biomarkers to elucidate the mechanisms by which transfusion of specific types of RBC products lead to adverse patient outcomes. Given its procoagulant nature, potential outcomes linked to cfDNA in RBC units that could be studied prospectively include cardiovascular events such as myocardial infarction, cerebrovascular accidents, or venous thromboses such as deep vein thrombosis and pulmonary embolism. In addition, presence of mtDNA in platelet concentrates has been linked to nonhemolytic transfusion reactions [27]. A prospective study may be able to confirm this finding in RBC units.

In conclusion, our study found that red blood cells processed by the WBF method and red blood cells with a shorter duration of storage were associated with increased concentrations of cfDNA. These findings are consistent with the clinical observations that fresh WBF blood may be associated with increased mortality in transfused patients. Further studies are required to confirm these observations and to understand the pathobiology.

## Figures and Tables

**Table 1:** Differences in cfDNA between red blood cells (RBCs) processed by whole blood filtration (WBF) and red cell filtration (RCF).

	RBCs processed by WBF	RBCs processed by RCF	*p* value
cfDNA by PicoGreen (mean ng/mL ± SD)	1.08 ± 0.90 (*n* = 44)	0.50 ± 0.77 (*n* = 73)	0.0010
cfDNA by Spectrophotometry (mean *µ*g/mL ± SD)	3.57 ± 1.99 (*n* = 47)	3.28 ± 1.28 (*n* = 73)	0.088

**Table 2:** The Interaction between storage duration and processing method on differences in cfDNA.

Assay	Red cell storage duration	Method of whole blood processing		*p* value
		Whole blood filtration (WBF)	Red cell filtration (RCF)	
cfDNA by PicoGreen (mean ng/mL ± SD)	Fresh (≤14 days)	1.16 ± 1.14 (*n* = 15)	0.37 ± 0.77 (*n* = 44)	0.0093
	Older (>14 days)	1.04 ± 0.78 (*n* = 29)	0.68 ± 0.74 (*n* = 29)	0.33

cfDNA by spectrophotometry (mean *µ*g/mL ± SD)	Fresh (≤14 days)	4.15 ± 2.47 (*n* = 16)	3.63 ± 1.25 (*n* = 44)	0.67
	Older (>14 days)	3.27 ± 1.65 (*n* = 31)	2.75 ± 1.15 (*n* = 29)	0.57

**Table 3:** Differences in cfDNA between fresh and older blood.

Assay	Duration of red blood cell storage		*p* value
	Fresh blood (≤14 days)	Older blood (>14 days)	
cfDNA by PicoGreen (mean ng/mL ± SD)	0.57 ± 0.93 (*n* = 59)	0.86 ± 0.78 (*n* = 58)	0.33
cfDNA by spectrophotometry (mean *µ*g/mL ± SD)	3.77 ± 1.66 (*n* = 60)	3.02 ± 1.44 (*n* = 60)	0.0031
